# A Descriptive and Phenomenological Exploration of the Spiritual Needs of Chinese Children Hospitalized with Cancer

**DOI:** 10.3390/ijerph192013217

**Published:** 2022-10-14

**Authors:** Qi Liu, Ka-Yan Ho, Katherine-Ka-Wai Lam, Winsome-Yuk-Yin Lam, Eileen-Hui-Lin Cheng, Shirley-Siu-Yin Ching, Frances-Kam-Yuet Wong

**Affiliations:** School of Nursing, Hong Kong Polytechnic University, Hong Kong SAR, China

**Keywords:** spiritual well-being, pediatric oncology, psychological health, symptoms, Chinese

## Abstract

Spiritual well-being is the fourth dimension of health, as equally important as physical, mental, and social well-being. The shadow of death associated with cancer triggers children to explore their personal values, meanings, and life goals throughout the illness trajectory, enabling them to identify their unique spiritual needs. Chinese children are generally non-religious, unlike Western children, which affects their spiritual needs. To address the literature gaps, we applied a qualitative, descriptive, phenomenological approach for exploring the spiritual needs of Chinese children hospitalized with cancer. Purposive sampling was conducted in two public hospitals with special wards for pediatric oncology patients in Hunan Province, China. Consequently, 22 children, hospitalized with cancer, were recruited and individually interviewed using a semi-structured interview format. We conducted a thematic analysis of the interview transcripts. Four important themes were identified: the need for self-exploration, inner needs, need for a connection with others, and need for a connection with gods, supernatural powers, and fictional characters. We found that culture significantly influenced the spiritual needs of Chinese children with cancer. Hope was a key factor motivating the children to continue cancer treatment. To address their unique spiritual needs, culturally specific interventions should be developed and incorporated into their care to enhance their spiritual well-being.

## 1. Introduction

Cancer is a leading cause of death among children worldwide, with approximately 400,000 children and adolescents being diagnosed with cancer every year [1]. In China, age-standardized childhood cancer incidence and mortality rates have been estimated at 87.1 and 36.3 per million, respectively [2]. New and advancing treatment options have significantly reduced the mortality rate of childhood cancer [3]. However, despite the improved chances of survival, as a life-threatening disease, cancer exposes children to potential losses, feelings of uncertainty about the disease, and a heightened sense of mortality and vulnerability [4]. All of these experiences trigger their exploration of personal value, meaning, and life goals throughout the illness trajectory, which, in turn, affects their spiritual well-being [5,6].

The World Health Organization defines spiritual well-being as the fourth dimension of health, as equally important as physical, mental, and social well-being [7]. By definition, spiritual well-being is a state that enables an individual to handle their daily activities, which enhances their awareness of their own potential, meaning, and life goals, and thus the achievement of happiness [8]. In addition, spiritual needs are defined as individuals’ needs and expectations in the pursuit of meaning, purpose, and value in their lives, and a connection to the self, others, the significant, or the scared [9].

Spirituality is a complex concept, which seems to be frequently linked to religiosity [10]. However, these two concepts are distinct [11]. Whereas spirituality foregrounds transcendence and focuses on meaning and purpose [12], religiosity can be considered as the means for identifying meaning and purpose by engaging with a formal religious group, doctrines, and traditions [11]. Human beings have an urge to seek meaning without necessarily holding a particular set of religious beliefs [12]. The complexity and ambiguity of the concept of spirituality compounds the difficulty of assessing spiritual needs [13]. Nevertheless, the spiritual needs of adult cancer patients can be broadly categorized within several domains. The most commonly reported domains are needs relating to find meaning and purpose, love, peace, belonging and connectedness, and forgiveness [14]. There is mounting evidence that the fulfillment of spiritual needs is associated with various positive patient outcomes [15,16,17]. Several studies also suggested that fulfilled spiritual needs for childhood cancer patients are sources of strength that assist patients in coping with the disease, helping them find meaning and peace throughout the course of the disease, notwithstanding the devastating symptoms that they endure [18,19,20]. Consequently, their anxiety and depressive symptoms are reduced, and their quality of life is ultimately improved [21,22].

Despite the importance of spirituality in the field of oncology, research in this area, and especially on children, is at a nascent stage. Few studies have explored the spiritual needs of children with cancer. One example in a qualitative study of 60 children aged 6–17 years with advanced cancer in the United States [23]. This study found that the spiritual needs of this population group were relational in nature, mostly involving praying to a god and/or a higher power to request help to feel better. Another study by Zeighamy and Sadeghi revealed four spiritual needs among adolescents with cancer who lived in Iran, which included the need to have a relationship with God, a relationship with the self, a relationship with others, and a relationship with the environment and nature [24]. Spiritual needs are particularly pertinent for children with cancer because the experience of living under the shadow of death prompts them to self-reflect and search for meaning and purpose in their lives [18]. It also prompts them to readjust their relationships with others in spite of the pain and suffering that they endure because of the disease and its treatment [22]. Childhood cancer patients who are provided with appropriate support will identify the positive aspects of this negative experience and perhaps view life as being more meaningful [25]. Although some studies have described the spiritual needs of children with cancer, they were conducted in the United States and Iran, thus limiting their generalizability to the Chinese population. The latter, though mostly non-religious, is strongly influenced by Confucianism, Taoism, and Buddhism [26,27]. Conner and Eller also pointed out that ethnicity plays an important role in determining spiritual needs [28]. To address gaps in the existing literature, we applied a qualitative approach to explore the spiritual needs of Chinese children hospitalized with cancer.

## 2. Materials and Methods

### 2.1. Study Design

We applied a descriptive, phenomenological approach in this study. This approach was chosen because it is commonly used to provide detailed descriptions of individuals’ lived experiences [29].

### 2.2. Participants and Setting

Purposive sampling was used to recruit participants from two public hospitals with special wards for pediatric oncology patients in Hunan Province in south-central China. Specifically, we set out to recruit childhood cancer patients with different types of cancer (i.e., solid and non-solid tumors) and obtained their time since diagnosis (i.e., <6 months, 6–12 months, 1–2 year, and >2 year), gender (i.e., male and female), and age (i.e., 8–12 years old and 13–17 years old). The rationale for choosing these two hospitals was that they offer advanced medical technology, making them the preferred choice for people in this province seeking cancer treatment for their children. The recruitment of participants at these two hospitals ensured the representation of diverse clinical backgrounds.

To be eligible for inclusion in the study, participants had to be aged 8–17 years, diagnosed with any type of cancer and treated at any point after their diagnosis, knowing that they had cancer, and be able to speak Chinese. We excluded individuals with identified cognitive and/or behavioral problems, which affected their verbal communication. Although we appreciate that children younger than 8 years also have spiritual needs, they may not have possessed the adequate skills to articulate their thoughts given their immature cognitive development [30]. Therefore, we only targeted children aged 8 years or above for the interviews.

### 2.3. Data Collection

We obtained ethical approval for the study (HSEARS20220127001) from the Institutional Review Board of the Hong Kong Polytechnic University. This study has also been registered at ClinicalTrials.gov (NCT05371236).

The head nurses in the pediatric oncology wards identified eligible children for this study and referred them to the research team. A research team member (QL) then approached the parents of these children and explained the purpose of the study to them in detail. If the parents permitted their children to join, the children were then asked if they were willing to participate in the study. After obtaining written parental consent, we scheduled the interviews.

All interviews were conducted by QL (a female, Chinese registered nurse and PhD candidate who had received training in qualitative methods by the senior research team members) at the patients’ bedsides. Each room only contained two beds with a distance of two and a half meters apart. The two beds were also separated by sound-deadening curtains. Prior to the semi-structured interviews, a pilot was done, and the results showed that conversations were unable to be transfer outside of the curtains once the curtains were drawn. The patients’ privacy was therefore ensured. Because the patients were minors, they were given the option to have their parents and/or caregivers present during the interview. The interviews were conducted using a semi-structured interview guide (see Supplementary File S1) developed by an expert panel comprising a professor (FKYW), an associate professor (SC), three assistant professors (KYH, WL, and CE) and a research assistant professor (KKWL). Each of these individuals have extensive experience of working with pediatric oncology patients or conducting research in this field. To ensure content validity, the interview guide was critically reviewed by two pediatric oncology nurses prior to conducting the interviews, with no amendment made.

Prior to the interviews, the researcher (QL) did not have any relationship established with the participants and their parents. This is a common practice in qualitative studies in this population group [31,32,33,34]. In addition, all participants and their parents were told that QL was a PhD student from a University in Hong Kong.

All of the interviews, which lasted between 20 and 80 min, were initiated with an open-ended, non-directive question: “How are you feeling today?”. This question was followed by more specific questions on the meaning of spirituality. Probing questions were asked to encourage the participants to share more information. Some examples include: “Which experience in your cancer journey makes you feel fulfilled?” and “What is the impact of spirituality on your cancer journey?”. The interview questions were all tested in three childhood cancer patients with different ages (i.e., 8, 11, and 15 years old) to make sure that the questions were understandable for our targeted participants, especially those who were at younger ages. All of the interviews were digitally recorded. In addition, field notes were written by QL to document key observations and her interactions with the participants in the interviews. According to the safety protocol, participants who were found to be potentially suicidal or at risk of self-harm would be referred to the nurse in charge on the ward who would inform the physicians to conduct a further clinical assessment.

Data saturation was considered to be reached when two coders noted repetition in the participants’ responses and no new subthemes or themes related to spiritual needs were identified [35]. Data saturation was achieved after interviewing 22 participants. Although we informed the participants that interviews could be conducted more than once when deemed necessary, all participants said their perceptions and feelings were adequately expressed. In addition, we did not identify any ambiguity when reading the transcripts. Hence, repeat interviews were not done.

### 2.4. Data Analysis

The data collection and analysis were performed simultaneously. A thematic analysis was performed on the interview transcripts [36]. After the interviews, the recordings were immediately transcribed, verbatim, in Chinese to capture any nuances of expression unique to the dialect. Subsequently, two researchers (QL and KYH) listened to the recordings repeatedly until they obtained a good sense of the overall content. Afterwards, they each read the transcripts line by line to identify any meaningful units relating to spiritual needs. Throughout the process, they compared the identified meaningful units for relevance until they reach a consensus. These units were then condensed into codes according to their similarities, and subthemes and themes were then identified. Since we used the descriptive phenomenology which emphasized the objectivity of the participants’ experiences [37], our data analysis was not inspired by other research and instruments. To maintain the objectivity of the analysis, the researchers who analyzed the data (QL and KYH) were asked to write their pre-existing beliefs and perceptions explicitly before the data collection, to ensure that any preunderstandings or presumptions could be set aside as far as possible. Considering the literacy level of the participants, the researcher did not return the transcripts and findings for them to comment. This is also a common practice for other qualitative studies conducted in childhood populations [31,32,38,39]. However, we sent the qualitative findings to one qualitative expert, who was not involved in the data analysis, for validation.

## 3. Results

### 3.1. Demographic and Clinical Characteristics of Participants

From February 2022 to May 2022, we have approached a total of 25 childhood cancer patients. Of them, three refused to participate. The reasons included a lack of interest (n = 2) and hesitation to talk about their feelings (*n* = 1). Hence, we recruited and interviewed a total of 22 children hospitalized with cancer. Four participants aged 9–11 chose to have their mothers present during the interview. The mean age of our participants was 12.4 years (SD = 3.5). Approximately 50% were female. The majority were diagnosed with leukemia, with lymphoma being the second most common cancer. Most of the children were diagnosed within a year of commencing the study and reported no religious beliefs. Table 1 shows the demographic and clinical characteristics of our participants.

### 3.2. Spiritual Needs of Children Hospitalized with Cancer

Four themes were derived from the semi-structured interviews. Supplementary File S2 presents the themes, subthemes, and illustrative quotes from the interviews.

#### 3.2.1. Theme 1. Need for Self-Exploration

##### Subtheme 1. Why Do I Have Cancer?

Throughout the disease trajectory, children with cancer wanted to know the reason why they, and not others, were sick (*n* = 15). As Respondent C11 (female, aged 10, lymphoma, 19 months since diagnosis, no religion) stated, “I often stare at the ceiling and think about why I am sick”. The respondents posited various causes for their disease. For example, some (*n* = 6) thought that karma of some kind was the underlying reason for their cancer. For example, Respondent C4 (female, aged 16, osteosarcoma, 24 months since diagnosis, no religion) observed: “I have bullied other children before. That’s why now I am punished”. Other respondents attributed their disease to diet. Thus, Respondent C2 (female, aged 9, leukemia, 5 months since diagnosis, Buddhism) stated: “I was a picky eater. I didn’t like to have meat, and so I didn’t have adequate protein. This weakened my immunity system. That’s why I got sick”.

##### Subtheme 2. What Does Cancer Mean in Their Lives?

Apart from seeking the reasons why they had cancer, the respondents also expressed mixed perceptions regarding their experiences of cancer. Some respondents (*n* = 9) viewed cancer as a disaster, which was destroying their entire families. As Respondent C15 (female, aged 14, leukemia, 10 months since diagnosis, no religion) noted: “My parents always argued and even fought with each other for money after I got sick. I don’t want to treat the disease”. Respondent (C12, male, aged 12, osteosarcoma, 29 months since diagnosis, no religion) expressed a similar view:

My parents borrowed a lot of money for my cancer treatment. They have also stopped working because they want to take care of me. Even if I recover from cancer, we still need to work very hard to pay off the debt. I don’t want to create a burden for my parents.

Conversely, some respondent (*n* = 7) perceived some positive aspects to having cancer. In particular, they regarded cancer as a challenge and believed that they would become stronger after surviving the disease. Some respondents (*n* = 4) also stated that this experience made them aware that time was precious, so they would not waste any more time on meaningless pursuits. This view was expressed as follows by Interviewee C10 (male, aged 16, lymphoma, 7 months since diagnosis, no religion): “I promised myself that I would perform better in the examination if I recover. After having the disease, I understand that time is precious. I will definitely study harder if I am able to go to school again”.

##### Subtheme 3. What Is Death?

Some respondents (*n* = 6) mentioned the topic of death. Specifically, they viewed death as an unavoidable topic having observed that some hospitalized children passed away because of the disease. Sometimes, they also overheard doctors and nurses telling parents that there was no chance of a cure for their children and therefore they did not have to stay in the hospital. All of these events triggered them to think about what death is. However, some respondents (*n* = 3) said that they seldom discussed this issue with their parents or with healthcare professionals because they always emphasized that the disease could be cured. Therefore, the respondents felt uncertain and could only conjecture about what would happen during the dying process. The following comment by Respondent C11 (female, aged 10, lymphoma, 19 months since diagnosis, no religion) illustrates this perspective:

I want to know what will happen when I die. [Interviewer: What do you want to know?] Um … Will I feel pain when I die? I also overheard someone say that people who are dying will see a bright light. I want to know whether it is true. I hope there is someone who can tell me so I don’t have to guess myself.

Some other respondents (*n* = 3) said they knew what death was because they had previously talked to their parents about the issue. They also felt no fear throughout the illness trajectory because they were prepared for the worst. As Respondent C15 (female, aged 14, leukemia, 10 months since diagnosis, no religion) put it:

The worst [that can happen] is that my disease cannot be cured and I will die. I have always expected this. [Interviewer: Why?] Because I don’t have to always think about this issue. I am prepared for the worst. There is nothing worse than that.

All of the respondents who had previously thought about death regarded it as an end. They also felt that nothing would be left behind after death. Some said they did not fear death, but they also did not want to lose their connections with their loved ones, namely their parents and friends. Respondent C7 (female, aged 11, lymphoma, 8 months since diagnosis, no religion) remarked: “I am not afraid of death. I just worry that I won’t be able to see my mom and dad anymore. They are important to me”.

#### 3.2.2. Theme 2. Inner Needs

##### Subtheme 1. Need for Peace

A majority of respondents (*n* = 17) mentioned that they desired peace, that is, a sense of tranquility. Although some were hospitalized to undergo cancer treatment with a high probability of being cured, they said they did not feel any peace because they were physically and psychologically tortured by the disease and by persistent negative thoughts. The respondents described feelings such as “being burned alive,” “wanting to jump out of a window,” and “being cramped”. Respondent C9 (male, aged 17, colorectal cancer, 21 months since diagnosis, no religion) elaborated on these feelings during the interview:

I have been sick for almost two years. My mom always encourages me, [saying] that I don’t have to be depressed because I will get better after [the] treatment. However, she doesn’t really understand. I cannot stop thinking about my disease. [Interviewer: What exactly do you think about?] Many things.…Will I recover? What will happen tomorrow? Whether the medication will work. I also feel anxious when waiting for the lab reports. I feel like I’m in prison.

Respondent (C5, male, aged 15, leukemia, 3 months since diagnosis, no religion) expressed the following view: “Even when I was extremely sick, I couldn’t stop thinking about the disease. Sometimes, I preferred to jump out of the window to stop everything instead of being tortured”. To gain a sense of peace, some respondents said that they applied various methods to distract themselves, including drawing, making handicrafts, and talking to others. However, some of the respondents stated that they preferred to stay alone and to deal with their negative thoughts alone. For example, Respondent C18 (female, aged 13, lymphoma, 30 months since diagnosis, no religion) stated: “I usually lie down on my bed and cry silently. I don’t want to bother anyone. I have already brought a lot of trouble to them”.

##### Subtheme 2. Need for Hope

Apart from peace, many respondents (*n* = 18) mentioned that they desired hope, which they regarded as a key motivator and source of strength, helping them to overcome the pain and suffering that resulted from cancer and its treatment. As respondent C18 (female, aged 13, lymphoma, 30 months since diagnosis, no religion) stated, “I always tell myself that I can’t die. I am still young. I still have a lot of things ahead of [me in] my life. That’s why I tolerate all of this [cancer and its treatment] even [though it is] so painful”. Hope, according to the respondents, could be influenced by things that happen in the short term or the long term. Short-term influences mentioned by most respondents included feeling much more hopeful when they knew that there was an improvement in the clinical data.

This view was expressed by Respondent C22 (male, aged 9, leukemia, 7 months since diagnosis, no religion) as follows: “I pay attention to any change in my white blood cell count. When I know the count is stable, it seems that the disease is under control. Then, I feel there is a hope in my life again”. When speaking about the long term, most respondents mentioned that they had a lot of dreams and goals to accomplish in their lives, which included going back to school, being able to eat what they wanted, and traveling and career aspirations as adults. All of these aspirations became their hopes and drove them to fight continuously to overcome the disease. Respondent C19 (female, aged 16, leukemia, 13 months since diagnosis, Christian) stated: “I believe that I can go back to the school very soon. All my classmates are studying, and I am the one in the hospital. They are waiting for me. I will recover”.

#### 3.2.3. Theme 3. Need for Connections with Others

##### Subtheme 1. Able to Express Themselves to Others

Several respondents (*n* = 8) said that they would like to talk to their loved ones, especially their mothers, when experiencing emotional distress because they gained considerable relief after sharing. As Respondent C7 (female, aged 11, lymphoma, 8 months since diagnosis, no religion) remarked, “My mom told me that I must tell her when I am feeling unhappy. I did. I felt much happier after saying it (the unhappiness) out”. Apart from expressing themselves with their mothers, some respondents (*n* = 5) said that they wanted to share their distress with their good friends. However, they said it was difficult to do this, as their friends were busy with school “stuff”. Some respondents (*n* = 3) mentioned that they would like to talk to other children whose experiences were similar because these children would be able to understand how they felt. Some respondents (*n* = 3) also stated that they wanted to share their distress with the doctors and nurses, who as professionals, would not judge their experience.

Although some respondents said that they wanted to share their distress with their parents, they hesitated to do so because they did not want to burden their parents with more troubles (*n* = 6). This point was illustrated by Respondent C12 (male, aged 12, osteosarcoma, 29 months since diagnosis, no religion) as follows: “They [my parents] are very upset about my disease. How can I tell them that I am unhappy? I don’t want to make them more upset because of me”. Some respondents also said that they wanted to pretend they were strong to comfort their parents and did not therefore mention their distress. Respondent C9 (male, aged 17, colorectal cancer, 21 months since diagnosis, no religion) gave the following explanation: “After I was diagnosed with cancer, my mom didn’t eat and drink. She is completely fed up. That’s why I need to be strong even when I am sad. I am the only one who can comfort her”. Moreover, some respondents felt that their parents did not understand how they felt and so they did not want to share their feelings: “I am the one who undergoes surgery. I am the only one who feels the pain. No one can understand what I have endured, not even my parents. I would rather keep quiet” (Respondent C14, male, aged 13, kidney tumor, 10 months since diagnosis, no religion).

##### Subtheme 2. Feeling Supported by Others

During the interviews, all of the respondents (*n* = 22) expressed the need to receive support from those around them, including their parents, friends, and healthcare professionals. This support made them feel loved and motivated them to continue the treatment. In addition, the support could manifest in different ways, such as spending time together, sharing snacks, and hearing some encouraging words. Some respondents (*n* = 8) noted that even trivial things were enough to make them feel happy and supported, as illustrated by the following statement:

I am so happy when doctors and nurses come to see me every morning. I know they are busy. But even just some greeting words, like good morning, how are you … this cheers me up. I know they are supporting me. (Respondent C14, male, aged 13, kidney tumor, 10 months since diagnosis, no religion).

Although all of the respondents mentioned the need for support, some (*n* = 6) said that they wanted to be treated as adults and preferred to hear the truth:

I know they [parents] want to cheer me up. I know. But I want to hear something real. They think I am a kid. Yes, I am a kid, but I can differentiate between what is real and what is not real. They always tell me that I will recover. However, I know my condition. I prefer that they tell me the truth rather than lie to me. (Respondent C21, female, aged 13, osteosarcoma, 9 months since diagnosis, no religion)

Despite stating that their friends and classmates were important sources of support, the majority of respondents (*n* = 18) said they were unable to maintain these friendships in the same way as previously. Specifically, some said (*n* = 7) they have greatly reduced contact with their friends because of their medical condition. Respondent C22 (male, aged 9, leukemia, 7 months since diagnosis, no religion) explained this situation as follows:

At the very beginning, I used to play with them [my friends]. However, I got fever every time. My mom said [that] my immunity was not good because of the treatment, so I couldn’t go to any crowded place. So, I now play alone at home.

Some respondents (*n* = 5) also stated that they had tried to keep up their friendships with their classmates. However, they had little in common and so they ran out of conversation topics quickly. Respondent C19 (female, aged 16, leukemia, 13 months since diagnosis, Christian) made the following remark:

When I was diagnosed with cancer, my classmates used to visit me. But every visit was the same; asking me how I felt and whether I was fine. They are all in school and I am the only one staying in the hospital. We have little in common. That’s why our conversation ended quickly during every visit. Later on, we drifted apart and stopped contacting each other.

Some respondents (*n* = 6) even stated that they did not want to meet their friends as they did not want to be teased about their changed appearance, particularly their loss of hair. Respondent C7 (female, aged 11, lymphoma, 8 months since diagnosis, no religion) stated: “My hair was very short. I am a girl. I worried that they [my friends] would make fun of me because of my odd looks. I don’t want to find them”.

##### Subtheme 3. Wanting to Be Helpful to Others

Although most respondents noted that they had to be taken care of by others throughout the disease trajectory, some (*n* = 5) said that they wanted to do something for others. Thus, they felt that their lives were still meaningful. Respondent C19 (female, aged 16, leukemia, 13 months since diagnosis, Christian) shared the following experience:

There was a boy in the ward, who did not want to talk and eat. So, I talked to him and played games with him. Later on, he became happier and more willing to talk. We also ate together. I felt very happy. [Interviewer: Why?] Because I found that I was not useless. It [my life] is still meaningful.

#### 3.2.4. Theme 4. Need for Connections with Gods, Supernatural Powers, and Fictional Characters

##### Subtheme 1. Feeling of Being Protected

A few respondents (*n* = 4) stated that religious beliefs played a very important supporting role, helping them to cope with the disease. When they were scared or upset, they would engage in religious practices to gain power from the gods. They believed that they were protected, and this feeling gave them considerable relief. Respondent C2 (female, aged 9, leukemia, 5 months since diagnosis, Buddhism), whose mother was Buddhist, stated: “I chant the scriptures with my mom every morning and night. The blessing from Buddha makes me feel relieved. The Buddha will be with me and protect me. I don’t feel scared when I chant”. Those respondents (*n* = 5) who lacked religious beliefs sought protection from other sources, including the zodiac and ancestors:

I was born in the year of the rabbit [in the Chinese zodiac]. I have a golden bunny necklace, which I always wear. The bunny is my protector. When I am scared or upset, I will hold the bunny. I recall that my mother was unable to accompany me when I had a bone marrow biopsy. But I knew the bunny was with me and accompanied me, so I was not scared. (Respondent C13, female, aged 9, lymphoma, 4 months since diagnosis, no religion).

##### Subtheme 2. Learning from Fictional Characters

Apart from seeking protection from gods and supernatural powers, some of the respondents (*n* = 5) stated that fictional characters in comics, cartoon films, movies, and games were brave and overcame challenges. Therefore, they wanted to learn from these characters and be strong so as to fight the disease. This view was illustrated by Respondent C16 (male, aged 8, lymphoma, 11 months since diagnosis, no religion) as follows:

My favorite mobile game is Ninja. I started playing this game after I got sick. This game helps me a lot. [Interviewer: Why?] I like the main character in this game, who is called Ninja. He is a Japanese hero who can’t be killed by any enemy. I am also Ninja, and I will not be killed by cancer”.

## 4. Discussion

Spiritual well-being is an important aspect of health. Like children in the United States and Iran who are afflicted with cancer [23,24], our Chinese respondents expressed various spiritual needs during their hospitalization. These needs included the need for self-exploration; inner needs; the need for connections with others; and the need for connections with gods, supernatural powers, and fictional characters. However, given cultural influences, our Chinese respondents interpreted their needs differently from Western and Middle Eastern children.

During the self-exploration process, a key question raised by our respondents concerned the cause of their cancer. Similar to children in the West [22,40], most of our respondents attributed their disease to external factors. However, differing from children living in Western countries, who mostly attribute cancer to the lack of God’s protection [22,40], several of our respondents attributed cancer to bad karma. Karma is a core concept in Buddhism, which has deep roots in China and influences people’s thoughts, irrespective of their religion [41]. Karma refers to a cycle of cause and effect [41]. Severe illnesses, such as cancer, are conceived as a punishment for a misdeed in the present life or in a previous life and/or as an outcome of the ancestors’ collective moral failure [42]. Therefore, unlike Western children with cancer who feel angry with God, some Chinese children and their families may feel ashamed of having the disease and cover up their condition from their friends and relatives [43,44].

Another question raised by our respondents in the self-exploration process concerned with the meaning of having cancer in their lives. Consistent with the findings reported in the literature [5], we found that cancer was viewed by some respondents as a challenge, which could lead to post-traumatic growth. However, most respondents perceived themselves as a financial burden to their families. Some even denied the value of their existence and wanted to give up their treatment. The influence of filial piety, one of the essential virtues in Confucianism [45], could account for this perception. This concept places a strong emphasis on children’s obedience and their attempts to do good, thus making their parents proud of them and avoiding bringing trouble upon their parents [46]. In addition, despite the introduction of a series of medical insurance schemes in China, catastrophic health expenditure remains a serious problem for households with cancer patients [47,48]. In one cross-sectional study of 242 households living with pediatric leukemia patients in China, 43.3% were found to have medical expenses that they were unable to pay for. Of these households, 97.1% obtained financial support from relatives and friends and 44% incurred debt [49]. Under such circumstances, cancer could lead to self-blame on the part of the patient rather than facilitating post-traumatic growth.

The second category of spiritual needs was inner needs, which included the needs for hope and peace. Our findings are in line with those reported in Western studies, namely that hope and peace are fundamental human needs linked to survival [50] and are mutually reinforcing, with higher levels of hope resulting in higher levels of peace [51]. Carson et al. distinguished two types of hope [52]. The first type is eternal hope, which is detached from personal desires and resides in God [52]. Another type is existential hope, which focuses on an individual’s future orientation [52]. In this qualitative study, only examples of existential hope, such as being able to return to school, traveling, and career aspirations were mentioned by the respondents; eternal hope was not discussed. A possible explanation is that China is one of the least religious countries in the world, with only 15% of its population having religious affiliations [53]. Our respondents’ hopes were therefore grounded in their imagination of future outcomes and goals, as opposed to relationships with God.

The third spiritual need for Chinese children with cancer was the need for connections with others. This spiritual need has been frequently reported in Western studies [54] and appears to be a key source of support for coping with various chronic diseases [55]. Some of the respondents whom we interviewed reported that they wanted to express their suffering and emotional distress to their loved ones. In addition, they mentioned that the feeling of being supported by others motivated them to continue their cancer treatment, notwithstanding their suffering and distress. These findings add to the existing literature, revealing that expression allows individuals to rationalize and validate their own emotions [56]. At the same time, the care of those surrounding the individual enhances their ability to handle stressors, such as cancer, resulting in better coping capabilities [19]. Our findings on the need to be helpful to others is indicative of a way of connecting with others. Indeed, a finding reported in the literature is that the fulfillment of this need can help individuals to overcome an end-of-life existential crisis centering on an inner conflict, characterized by feelings that life is meaningless [57]. Being helpful to others could heighten an individual’s general sense of purpose and meaning in life [58], which, in turn, could assist them to overcome an existential crisis, ultimately improving their spiritual well-being.

The last spiritual need was the need for connecting with gods, supernatural powers, and fictional characters. Unlike children in the West who mostly seek shelter within their religious communities and engage in religious practices [59], a majority of our respondents expressed the need to connect with ancestors and with the Chinese zodiac (known as Sheng Xiao) and were inclined to seek their protection. This finding can be similarly explained by the fact that most Chinese people are not religious, preferring to engage in multiple traditional customs handed down the generations [53]. For example, the Chinese zodiac entails a repeating cycle of 12 years. Each year is associated with one of the 12 animals. The birth year determines the zodiac sign, which can influence an individual’s fortune and personal traits [60]. Likewise, a substantial number of Chinese people believe that deceased family members may become ancestors in the supernatural world and continuously look after the family. The veneration of ancestors keeps them happy, and, in return, they will protect the family from misfortune [61]. These beliefs foster and influence faith [62]. Therefore, Chinese children with cancer seek protection from the Chinese zodiac and their ancestors instead of seeking protection from religious figures when they are scared and facing medical procedures and uncertain prognoses. Apart from ancestors and the Chinese zodiac, some respondents also reported that they derived their faith and courage from fictional characters in comics, cartoons, movies, and games, so as to cope with the disease. A possible explanation was that stories are important for children’s development and can be viewed as a medium for conveying values, beliefs, attitudes, and social norms [63]. When reading or listening to a story, children identify with the fictional characters with whom they relate and follow the events of the storyline [64]. Consequently, they become emotionally involved with the story and ultimately gain insights into how the characters solve their problems [65]. During this process, they are instilled with faith.

### 4.1. Implications for Future Research and Practices

Our findings have two major research implications. First, the spiritual needs of Chinese children with cancer are strongly influenced by culture. However, the existing tools that can be used to assess the spiritual well-being of children with cancer are geared to the Western cultural context and do not consider the Chinese cultural context. Therefore, adaptations must be made to the existing tools before they can be applied for accurately assessing the spiritual well-being of Chinese children with cancer. The 12-item Functional Assessment of Chronic Illness Therapy-Spiritual Well-Being (FACIT-Sp-12) instrument is widely used to measure spiritual well-being in individuals with cancer and other chronic illnesses [66]. This tool has been applied within an increasing number of studies to assess the spiritual well-being of Western children with various diseases, including cancer [54,67]. Therefore, future researchers can try to modify and refine the FACIT-Sp-12 according to our findings for use with Chinese children with cancer. Quantitative studies should also be performed to identify the important factors contributing to their spiritual well-being using the adapted FACIT-Sp-12 tool.

Second, the need for hope was found to be a key source of support for Chinese children with cancer to overcome the pain and suffering that resulted from the disease and to motivate them to continue the treatment throughout their cancer trajectory. As such, providing hope to children with cancer seems to be an appropriate and feasible strategy for addressing their spiritual needs. Hope-centered therapy has been widely used to enhance hope and to promote the spiritual well-being of adult patients with cancer [68,69]. This therapy stems from positive psychology, which aims to foster hopeful thinking through the identification of personal goals and the advancement of skills and mindsets to achieve these goals [70]. Notwithstanding the promising results in adult cancer patients, the application of hope-centered therapy within the pediatric oncology patient population is less common. To date, there is no evidence that hope therapy can promote hope and the spiritual well-being of children with cancer in the Chinese context. Therefore, a large-scale, randomized controlled trial should be conducted to determine whether hope-centered therapy is a culturally suitable intervention for improving hope and the spiritual well-being of Chinese children with cancer.

### 4.2. Limitations

Notwithstanding the originality of the study, there were several limitations that warrant attention. First, this study only aimed to elicit a general description of the spiritual needs of Chinese children with cancer. Hence, we did not apply theoretical sampling to determine whether the children’s spiritual needs could change and how they may do so depending on the illness trajectory. Second, because of ethical considerations, we did not interview those who were terminally ill, thus avoiding potential psychological distress caused by the interviews. This gap undermined the generalizability of our findings and their applicability to Chinese children with terminal cancer. Third, we did not completely distinguish mental health and social health issues from spiritual issues when reporting our findings. This is because, according to the previous literature, spirituality is a concept intertwining with both psychological and social health [71,72]. A complete separation was therefore not possible. As such, quotes related to mental and social health issues have been identified and extracted in our data analysis, as long as these quotes were relevant to illustrate the spiritual needs of children. Fourth, this study is an exploratory study to obtain a preliminary understanding about the spiritual needs of childhood cancer patients in China. Despite age, gender and religious affiliation may impact on their needs; this is out of our scope of study. Additionally, a larger sample size is needed to delineate the impact of age, gender, and religious affiliation on the patients’ spiritual needs [73], and hence it is not possible at this stage given the small sample size. However, future studies are recommended to do so to study such an impact.

## 5. Conclusions

This study addressed gaps in the existing literature by exploring the spiritual needs of Chinese children hospitalized with cancer. Their spiritual needs were categorized into four domains derived from qualitative descriptions: the need for self-exploration; inner needs; the need for connections to others; and the need for connections to gods, supernatural powers, and fictional characters. Culture was found to play a significant role in influencing the spiritual needs of Chinese children with cancer. Therefore, culturally specific interventions should be developed and implemented in the context of end-of-life care to improve their spiritual well-being.

## Figures and Tables

**Table 1:** Demographic and clinical characteristics of participants (*n* = 22).

Sex, *n* (%)	
Male	10 (45.5)
Female	12 (54.5)
Age, *n* (%)	
8–12 years	10 (45.5)
13–17 years	12 (54.5)
Diagnosis, *n* (%)	
Lymphoma	7 (31.8)
Leukemia	9 (42.9)
Osteosarcoma	3 (13.6)
Kidney tumor	2 (16.7)
Colorectal cancer	1 (4.5)
Time since diagnosis, *n* (%)	
<6 months	3 (13.6)
6–12 months	10 (43.5)
1–2 year	6 (26.1)
>2 year	3 (13.6)
Home religious affiliation, *n* (%)	
Buddhism	2 (9.1)
Christianity	2 (9.1)
No religion	18 (81.8)
Religious practice, *n* (%)	
Yes	4 (18.2)
No	18 (81.8)

## Data Availability

Data will be available upon reasonable request.

## Supplementary Materials

The following supporting information can be downloaded at: https://www.mdpi.com/article/10.3390/ijerph192013217/s1.
