# Supplementary material for: A Descriptive and Phenomenological Exploration of the Spiritual Needs of Chinese Children Hospitalized with Cancer
Source: Int J Environ Res Public Health. 2022 Oct 14;19(20):13217. doi: 10.3390/ijerph192013217 (PMC9602965; doi:10.3390/ijerph192013217)
Supplement: Supplementary file 1 [file ijerph-19-13217-s001.zip › Supplementary file S2.pdf]

Supplementary File S2. An abridged table of themes, subthemes and examples of quotes from the interviews

Themes	Subthemes	Examples of quotes
1. Self-exploration (N= 20)	1. Why do I have cancer? (N= 15)	I have bullied other children before. That's why now I am not punished. *Respondent C4* (female, aged 16, osteosarcoma, 24 months since diagnosis, no religion)
	2. What does cancer mean in their lives? (N= 16)	My parents borrowed a lot of money for my cancer treatment. They have also stopped working because they want to take care of me. Even if I recover from cancer, we still need to work very hard to pay off the debt. I don't want to create a burden for my parents. *Respondent C12* (male, aged 12, osteosarcoma, 29 months since diagnosis, no religion)
	3. What is death? (N= 6)	I want to know what will happen when I die. [Interviewer: What do you want to know?] Um … Will I feel pain when I die? I also overheard someone say that people who are dying will see a bright light. I want to know whether it is true. I hope there is someone who can tell me so I don't have to guess myself. *Respondent C11*(female, aged 10, lymphoma, 19 months since diagnosis, no religion)
2. Inner needs (N= 20)	1. Needs for peace (N= 17)	I have been sick for almost two years. My mom always encourages me, [saying] that I don't have to be depressed because I will get better after [the] treatment. However, she doesn't really understand. I cannot stop thinking about my disease. [Interviewer: What exactly do you think about?] Many things.…Will I recover? What will happen tomorrow? Whether the medication will work. I also feel anxious when waiting for the lab reports. I feel like I'm in prison. *Respondent C9* (male, aged 17, colorectal cancer, 21 months since diagnosis, no religion)
	2. Need for hope (N= 18)	I always tell myself that I can't die. I am still young. I still have a lot of things ahead of [me in] my life. That's why I tolerate all of this [cancer and its treatment] even [though it is] so painful. *Respondent C18* (female, aged 13, lymphoma, 30 months since diagnosis, no religion)
3. Connections with others (N= 22)	1. Able to express themselves to others (N= 19)	My mom told me that I must tell her when I am feeling unhappy. I did. I felt much happier after saying it (the unhappiness) out. *Respondent C7* (female, aged 11, lymphoma, 8 months since diagnosis, no religion)
	2. Feeling supported by others (N= 22)	I am so happy when doctors and nurses come to see me every morning. I know they are busy. But even just some greeting words, like good morning, how are you … this cheers me up. I know they are supporting me. *Respondent C14* (male, aged 13, kidney tumor, 10 months since diagnosis, no religion)

	3. Wanting to be helpful to others (N= 5)	There was a boy in the ward, who did not want to talk and eat. So, I talked to him and played games with him. Later on, he became happier and more willing to talk. We also ate together. I felt very happy. [Interviewer: Why?] Because I found that I was not useless. It [my life] is still meaningful. *Respondent C19* (female, aged 16, leukemia, 13 months since diagnosis, Christian)
4. Connections with gods, supernatural powers, and fictional characters (N= 14)	1. Feeling of being protected (N= 9)	I was born in the year of the rabbit [in the Chinese zodiac]. I have a golden bunny necklace, which I always wear. The bunny is my protector. When I am scared or upset, I will hold the bunny. I recall that my mother was unable to accompany me when I had a bone marrow biopsy. But I knew the bunny was with me and accompanied me, so I was not scared. *Respondent C13* (female, aged 9, lymphoma, 9 months since diagnosis, no religion)
	2. Learning from fictional characters (N= 5)	My favorite mobile game is Ninja. I started playing this game after I got sick. This game helps me a lot. [Interviewer: Why?] I like the main character in this game, who is called Ninja. He is a Japanese hero who can't be killed by any enemy. I am also Ninja, and I will not be killed by cancer." *Respondent C16* (male, aged 8, lymphoma, 11 months since diagnosis, no religion)
