# Supplementary material for: A Descriptive and Phenomenological Exploration of the Spiritual Needs of Chinese Children Hospitalized with Cancer
Source: Int J Environ Res Public Health. 2022 Oct 14;19(20):13217. doi: 10.3390/ijerph192013217 (PMC9602965; doi:10.3390/ijerph192013217)
Supplement: Supplementary file 1 [file ijerph-19-13217-s001.zip › Supplementaty file S1.pdf]

Interview guide

How are you feeling today?
Could you tell me what it was like when you discovered the disease?
What were you thinking after you knew you were sick?
How do you think of your disease?
How has your illness affected your life?
What is the impact of spirituality on your cancer journey?
Which experience in your cancer journey makes you feel fulfilled?
Who or what gives you the most support in coping with your sickness?
Would you talk your minds with others? Are there any worries that you don not talk about them your feelings? (If they say no)
What gives you a sense of hope?
What are you afraid of? Why do you have these fears?
How do you see your future?
Is there some kind of goal you want to fulfill or look forward to accomplishing?
Do you have a religion?
Do you believe there is a higher being (i.e., Buddha, God)? Do you pray?
Are you scared of dying (if death and dying are talked about in previous conversation)?
